# Onco-epidemiology of domestic animals and targeted therapeutic attempts: perspectives on human oncology

**DOI:** 10.1007/s00432-014-1664-9

**Published:** 2014-05-11

**Authors:** Alessandro Di Cerbo, Beniamino Palmieri, Gionata De Vico, Tommaso Iannitti

**Affiliations:** 1 Poliambulatorio del Secondo Parere, Modena, Italy; 2 Department of General Surgery and Surgical Specialties, University of Modena and Reggio Emilia Medical School, Surgical Clinic, Modena, Italy; 3 Department of Biology, University Complex of Monte Sant’Angelo, Naples, Italy; 4 School of Biomedical Sciences, University of Leeds, Leeds, UK

**Keywords:** Animal models, Comparative oncology, Spontaneous tumors, Translational oncology

## Abstract

The spontaneous tumor biology has been investigated with the support of animalists using animals as a preclinical model allowing translation of results in clinical practice. This review provides an insight into the field of comparative oncology. Evidence shows that companion animal health care is impressively growing in terms of development of new therapies and diagnostic tools, nutrition and disease prevention. However, even if most animal tumors might be a reliable model to study human carcinomas, many open questions, related to the opportunities to select and recruit new models in oncology, along with their legal and ethical implications, remain unanswered.

## Introduction

The growing market of animal health products may account for the potential revenue source of companion animal care. The animal health care has resulted in more than one billion sales in 2007 for Novartis, Pfizer and Heska (Kling 2007). The opportunity to carry out preclinical trials based on spontaneous tumors has been considered suitable to develop new effective drugs that could be quickly translated into clinical practice (De Vico et al. 2005; Porrello et al. 2006; Vail and MacEwen 2000). For instance, naturally occurring tumors in dogs share similarities with human cancer both in terms of histopathological features and biological behavior (Hahn et al. 1994). In the second half of the 1800s, a substantial parallel between the animal and human oncopathology had already been observed (Porrello et al. 2006). In 1937, evidence showed that spontaneous animal tumors were similar to their human counterpart and information retrieved from them might have clinical implications (Dobberstein 1937). In the early 1970s, the first World Health Organization (WHO) International Histological Classification of Tumors of Domestic Animals was published (Beveridge and Sobin 1974, 1976). It was followed by the TNM Classification of Tumors in Domestic Animals based on the guidelines of the TNM Classification of Malignant Tumors in Man (Owen 1980) showing that spontaneous canine and feline tumors are relevant models to study human cancers. We summarized such models in Table 1 according to the review from De Vico et al. (2005). Conversely, in vivo and in vitro models, based on rodents and cell lines, have displayed intrinsic limits, such as complexity of management (Schiffer 1997) and the experimental methods used to induce the pathology (Vail and Thamm 2004), which make them different from the human spontaneous diseases (De Vico et al. 2005; Moore and Siopes 2004).

**Table 1:** Summary of tumor features shared between animals and humans (Allen et al. 2002)

Model	Kind of tumor	Features
Canine/Feline	Non-Hodgkin’s lymphoma	Equates to intermediate and high-grade non-Hodgkin’s lymphoma in humans Immune system alterations seem to be associated with a higher incidence of non-Hodgkin’s lymphoma Is very sensitive to chemotherapy Currently used as a model for testing new chemotherapeutics and new forms of immunotherapy, as well as for the study of multiple drug resistance; it has been used as a relevant model for (1) developing hypoxic cell markers, (2) studying the effect of whole-body hyperthermia on the pharmacokinetics of systemic chemotherapy and (3) studying autologous bone transplantation
Canine	Soft tissue sarcomas	Similar to pathological appearance, clinical presentation and behavior of human soft tissue sarcomas Respond to radiation therapy and chemotherapy in a manner similar to human pathology Hemangiosarcomas are extremely similar to human hemangiosarcomas with respect to histological pattern and metastatic behavior
Canine	Osteosarcomas	Similar to human osteosarcomas with respect to the histology, metastatic behavior and clinical evolution of the disease
Canine	Oral malignant melanomas	They are chemoresistant and share many immunological targets with human oral malignant melanomas Considered a relevant model for developing new immunotherapeutic approaches for both dogs and humans Both in dogs and humans, human tyrosinase DNA vaccination is safe and potentially effective
Canine	Transitional cell carcinomas	Share histological appearance, biological behavior and response to therapy with invasive human transitional cell carcinomas Considered a useful model for testing new photodynamic therapy technologies and chemotherapeutic combinations
Canine	Mast cell tumors	Mutation of exon 11 of c-kit occurs in 30–50 % of advanced mast cell tumors and is similar to that usually occurring in 50–90 % of human gastrointestinal stromal tumors
Canine/Feline	Mammary carcinomas	Very similar to human breast cancers because of their hormonal dependence, spontaneous development in middle-aged to older animals, metastatic behavior toward regional lymph nodes and lungs, as well as adhesion molecules and neoangiogenesis patterns Abnormalities in the nuclear DNA content have been documented in both malignant and benign canine mammary tumors, but are more frequent in human mammary carcinomas Mammary neoplasia in dogs may be a good molecular model for developing new antineoplastic strategies involving cyclin D1 and cyclin-dependent kinases Feline mammary carcinoma shows age incidence, histopathology and pattern of metastasis similar to human breast cancer Feline mammary carcinoma shares common features with human inflammatory mammary carcinoma and human mammary carcinoma with osteoclast-like giant cells Epidermal growth factor receptor-2 (HER-2) and recepteur d'origine nantais (RON) overexpressions qualify feline mammary carcinoma as homologous to human breast carcinomas which have poor prognosis
Canine	Seminomas	Metastases occur only in a small percentage of cases, whereas human seminomas have a marked tendency to metastasize Have a histogenetic behavior similar to that of human seminomas
Canine	Transmissible venereal tumor	Is considered a promising model to study human Kaposi’s sarcoma Percutaneous inoculation and intraarterial transplantation of tumor fragments in the canine lung result in predictable patterns of tumor growth resembling the solitary pulmonary nodules and metastatic disease found in humans

## Searching criteria

We searched PubMed/MEDLINE using the keywords “spontaneous”, “animal”, “tumor”, “oncology” and “comparative” alone or in combination and cross-referencing among published papers. Selected papers from 1910 to 2013 were chosen on the basis of their content (evidence-based quality and reliability).

### Epidemiology of cancer in dogs

Spontaneous canine tumors, such as prostate carcinoma, chondrosarcoma, which accounts for approximately 10 % of all primary bone tumors in dogs (Beveridge and Sobin 1976), axial osteosarcoma, which accounts for 80 % of all primary bone tumors in dogs (Thompson and Pool 2002) and synovial cell sarcoma, have been extensively described representing valuable models to study carcinogenesis (Vail et al. 1994). Most canine prostate carcinomas affect both elderly sexually intact dogs and dogs which had undergone surgical castration after reaching sexual maturity. In 2005–2009, a pilot study evaluated the incidence of spontaneous tumors in dogs living in the neighborhood of Venice and Vicenza (Vascellari et al. 2009). Two thousand five hundred and nine out of 296,318 canine cases of neoplasia were diagnosed during the first three years. The estimated annual tumor incidence rate per 100,000 dogs was 282, with an equal distribution of malignant and benign tumors between male and female dogs. Moreover, in pure breeds, a twofold higher incidence of malignant tumors, with respect to mixed breeds, was observed. The incidence increased with age in both genders. Due to the dissimilar methodologies and variable reference populations of both European and North American veterinary cancer registries, the occurrence of spontaneous tumors in pet animals has been underestimated (Bonnett et al. 1997; Dobson et al. 2002; Dorn et al. 1968; Parkin and Muir 1992; Priester and McKay 1980). The Animal Tumor Registry of Genoa (Italy) retrospectively showed that, from 1985 to 2002, 70 % of all cancer cases in female dogs were located in the breast (Merlo et al. 2008). The overall incidence of cancer was 99.3 per 100,000 dogs per year in male dogs and 272.1 in female dogs. Among domestic animal tumors, spontaneous squamous cell carcinomas provided additional information about the pathology of oral cancer (Gardner 1996). As far as oral tumors affecting dogs are concerned, malignant melanoma accounts for the 4 % of all canine tumors (MacEwen et al. 1999). Canine malignant melanomas and human melanomas are treated similarly using surgery and/or fractionated radiation therapy and immunological therapies (Vail and Thamm 2004; Bergman et al. 2003). Therefore, canine malignant melanomas are suitable models for new immunotherapeutic protocols in humans (Vail and Thamm 2004). Dogs and cats also frequently develop squamous cell carcinomas (Dorn and Priester 1976; Strafuss et al. 1976). Skin and subcutaneous tissue neoplasms are the most frequently recognized neoplastic disorders in domestic animals and can be caused by prolonged and continuous exposure to sunlight (Madewell 1981). Alimentary tract cancer in dogs has lower incidence, with ductal and acinar carcinomas occurring most frequently in females than in males while intranasal tumors account for 1–2 % of all canine neoplasms (Priester 1974). Primary bone cancer is the second most often detected in dogs, and its main risk factors are ionizing radiations, chemical carcinogens and viruses (Madewell 1981). Moreover, preexisting bone defects and skeletal abnormalities increase the risk factors from 60 up to 185 times for large and giant breeds with respect to small dogs (Tjalma 1966). As to primary brain tumors, they account for approximately 2 % of all cancer in human adults (McKinney 2004) and for 0.01 % in dogs (LeCouteur 1999). Specifically, gliomas are more common among brachycephalic breeds of dogs, especially boxers, English bulldogs and Boston terriers (Hayes 1976). High incidence rates were also detected for non-Hodgkin’s lymphoma in bitches and for non-Hodgkin’s lymphoma and skin cancer in male dogs (Merlo et al. 2008). Additionally, the incidence rate of cancer increased with age ranging between 23.7 and 763.2 in bitches and 16.5 and 237.6 in male dogs aged ≤3 and >9–11 years. Hemangiosarcoma, which can affect both cats and dogs, involves the musculoskeletal system, and mean survival time in dogs affected by this condition ranges from 267 (Ogilvie et al. 1996) to 780 days (Ward et al. 1994), depending on the disease stage. The ovarian and epithelial tumors, although quite rare in domestic animals, have been reported mainly in cats, dogs and horses (MacLachlan 1987). A single unique gonadoblastoma tumor affecting human males or females has been observed in the testicle of a dog (Turk et al. 1981). Spontaneous testicular tumors, which are quite common in aged dogs, are mainly seminomas (solitary, unilateral, more frequent in the right testicle and with a reduced possibility to develop metastases, if compared with humans) (Kennedy et al. 1998), Sertoli cell tumors and Leydig cell tumors, which can coexist together along with seminomas (Nielsen and Kennedy 1990) and occur with the same frequency (Maiolino et al. 2004). Maiolino et al. suggested a possible correspondence between human spermatocytic seminomas and most canine seminomas in order to justify their low metastatic behavior, showing a potential predictive model for the development of a treatment protocol (Maiolino et al. 2004). Further, rare dog tumors are fibrosarcoma, rib, vertebral body and pelvis tumors (both in dogs and in cats) (Dernell et al. 1998; Pirkey-Ehrhart et al. 1995; Straw et al. 1992) and multiple myeloma (rare in cats and uncommon in dogs), which accounts for 8 % of all canine hematopoietic tumors and affects older dogs with no breed or sex predilection (Matus et al. 1986) and intracranial central neurocytomas (Russell and Burch 1959). Canine mast cell tumors, which account for 16–21 % in this species (Thamm and Vail 2001), display a molecular alteration in the proto-oncogene c-kit, which is involved in mast cell differentiation, proliferation, survival and activation (London et al. 1999). Specifically, the mutation of exon 11 of c-kit occurs in 30–50 % of advanced mast cell tumors and is similar to that usually occurring in 50–90 % of human gastrointestinal stromal tumors (Heinrich et al. 2002; London et al. 2003). This evidence emphasizes a parallel between human and canine mast cell tumors underlying the possibility of using the canine model to develop new beneficial therapeutics for both species (Pryer et al. 2003). Non-Hodgkin lymphoma, which accounts for 5 % of all malignant tumors and 83 % of all hematopoietic malignancies in dogs (Vail and Thamm 2004), affects mostly middle-aged, older dogs (German shepherds, boxers, basset hounds and Saint Bernards) (MacEwen and Young 1996) and both genders equally and corresponds to intermediate and high-grade non-Hodgkin lymphoma in humans. It is generally associated with a poor prognosis, and in 70–80 % of cases, it is B-cell mediated, whereas in 20–30 %, it is T-cell mediated (MacEwen 1995). Due to its sensitivity to chemotherapy, it is used as a model both for development of new chemotherapy drugs and multiple drug resistance studies (Vail and MacEwen 2000). Moreover, non-Hodgkin lymphoma has been used to develop hypoxic cell markers, study the whole-body hyperthermia effects and evaluate autologous bone transplantation (Vail and Thamm 2004).

### Epidemiology of cancer in cats

Spontaneous osteosarcomas account for more than 70 % of malignant bone tumors in cats (Thompson and Pool 2002; Brodey and Riser 1969). These tumors are relevant to human cancer biology, due to their relative resistance to chemotherapy (He et al. 2014) and widespread metastatic potential. Injection-site sarcomas in felines, usually fibrosarcomas, are caused by an intense inflammatory reaction or vaccination adjuvants (Carwardine et al. 2013) and have extensively been reviewed (Hauck 2003). They are highly locally invasive and metastasize in up to 23 % of the affected cases (Seguin 2002). Although aggressive surgical resection remains the gold standard therapy, chemotherapy and radiation can prolong the survival times (Hershey et al. 2000; Kobayashi et al. 2002). Uterine tumors, rare in cats, account for 0.29 % of feline neoplasms (Miller et al. 2003), and they include endometrial adenocarcinoma, mixed mullerian tumor, leiomyoma which occurs most frequently (MacLachlan and Kennedy 2002) and myxoid leiomyosarcoma (Cooper et al. 2006). Even if the etiology of nervous system tumors is not well understood, cats are commonly affected by meningiomas (Troxel et al. 2003). However, the biological behavior of such tumor, except for the anaplastic type, is generally considered benign in both humans (Louis et al. 2007) and common pets like dogs and cats (Motta et al. 2012). A pilot study evaluated the incidence of spontaneous tumors in cats living in the neighborhood of Venice and Vicenza (Vascellari et al. 2009). Four hundred and ninety-four out of 214,683 feline cases of neoplasia were diagnosed during the first three years. For all tumors, the estimated annual incidence rate in cats per 100,000 was 77. Furthermore, a 4.6-fold higher incidence of malignant tumors was observed in cats, if compared to benign pathology. Moreover, in pure breed cats, a twofold higher incidence of malignant tumors was observed and it increased with age in both malignant and benign pathologies, if compared with mixed breeds.

### Translation of experimental studies into the clinical setting

In 2009, Wells introduced the potential role of pets in offering a therapeutic value to humans (Wells 2009b, 2012). Previous reports emphasized the role of dogs and cats as preventers of ill-health showing that pet owners are healthier than non-owners (Parslow et al. 2005; Wells 2009a; Wilson 1991; Serpell 1991; Allen et al. 2002; Anderson et al. 1992; Baun et al. 1984; Dembicki and Anderson 1996; Friedmann et al. 1980; Katcher 1981; Katcher et al. 1983; Larson et al. 2010; Sebkova 1977; Siegel 1993; Vormbrock and Grossberg 1988). Furthermore, the ability of pets to facilitate human recovery has been explored (Larson et al. 2010). For instance, Friedmann et al. reported that pet owners were significantly more likely to be alive 1 year after a heart attack than non-owners (Friedmann et al. 1980) and dogs were stronger facilitators to recovery from ill-health than cats (Friedmann and Thomas 1995). Therefore, dogs may contribute indirectly to long-standing human physical health, which is of great importance, especially in immunocompromised patients (Hemsworth and Pizer 2006), possibly due to the increased physical activity which typically characterizes the ownership of a dog (Larson et al. 2010). Dogs have also been used as warning systems for cancer (Williams and Pembroke 1989), epilepsy (Brown and Strong 2001; Dalziel et al. 2003; Strong et al. 1999, 2002) and diabetes (Chen et al. 2000; Lim et al. 1992; McAulay et al. 2001). They have also been used as therapists in nursing homes (Crowley-Robinson et al. 1996; Fick 1993; Kaiser et al. 2002), visitors in pediatric hospital wards (Moody et al. 2002), assistants for the disabled (Davis et al. 2004; Fishman 2003; Sanders 2000), enhancers of psychological welfare (Raina et al. 1999) and rehabilitators for prisoners (Merriam-Arduini 2000; Strimple 2003).

## Discussion

Comparative oncology research has extensively relied on domestic animals (Antinoff and Hahn 2004; Hansen and Khanna 2004; Hershey et al. 2005; Nasir and Reid 1999; Seltenhammer et al. 2004; Withrow and Vail 2007). The Comparative Oncology Trials Consortium Program Infrastructure, founded in 2009 to design and execute clinical trials involving dogs affected by cancer and in collaboration with the drug manufacturing industry and nongovernmental groups interested in cancer drug development, aimed to answer biological questions which could inform the development path of novel agents for future use in human cancer patients in a timely and integrated manner (Gordon et al. 2009). The recent identification of the canine genome has provided evidence of strong similarities with humans (Lindblad-Toh et al. 2005; O’Brien and Murphy 2003; Ostrander et al. 2006) such as several cancer-associated gene families (Hoffman and Birney 2007), as well as the presence of the similar genetic cancer-associated molecular alterations (Haga et al. 2001; Setoguchi et al. 2001; Thomas et al. 2003). Tumor initiation and progression are also influenced by age, nutrition, sex, reproductive status and environmental exposures in both human and canine cancers (Bukowski et al. 1998; Hayes and Fraumeni 1977; Misdorp 1996; Mukaratirwa 2005; Olson 2007; Patronek et al. 1997). The lack of gold standards for the management of cancer in dogs and cats, as well as the high motivation of pet owners to seek out new options for its management, underlines the increasing need to evaluate novel therapeutics in these populations. In this ways, it is possible to assess the effectiveness of new agents when given alone or in combination with other therapies prior to the clinical testing of the drug.

## Conclusions

The enrollment of pet dogs in preclinical and clinical trials is now focusing on new anti-neoplastic drug research and development. Although regulation of animal research possesses guidelines such as the three Rs (Replacement, Reduction and Refinement) (Russell and Burch 1959), legal implications regarding the use of animal models for research purposes are still debated (Griffin 1998; Porrello et al. 2004). Although the existence of spontaneous pet tumors could be a reliable model for human cancers, many open questions, related to the opportunities to select and recruit new types of animal models in oncology and to their legal and ethical implications, remain unanswered. Unfortunately, the use of spontaneous animal models implicates several professional and ethical warnings with conflictory debates. In the recent years, researchers have also focused their exploration on a possible role of some dog species as early warning systems for cancer (Williams and Pembroke 1989), epilepsy (Brown and Strong 2001; Dalziel et al. 2003; Strong et al. 1999, 2002) and diabetes (Chen et al. 2000; Lim et al. 1992; McAulay et al. 2001). While some dogs have showed innate ability to recognize with smell the human cancers in vivo, others have been trained to perform this skill (Willis et al. 2004). From this overview, there is evidence that companion animal health care is impressively growing in terms of development of new therapies and diagnostic tools, nutrition and disease prevention (Kling 2007). Conclusively, we strongly encourage the animalists, veterinarians and clinical researchers to join us and support ethical observational studies on domestic and laboratory animal spontaneous cancer epidemiology, early prevention, diagnosis and treatment in order to detect and possibly eliminate alimentary and environmental factors which can also be cancerogenic for humans. In fact, the shorter life span of animals, compared with that of humans, can give a rapid overview of many clinical features which also characterize human cancer. Furthermore, the pooling of anecdotal veterinary reports on drugs or multimodal animal cancer treatments, protocols and outcomes, including surgical pathology, oncoimmunology and molecular biology investigations, will give outstanding contribution to the interspecies translational information with undoubted mutual interspecies benefit. The pet psychological and physical support to the fitness and wellness of cancer patients should be more deeply investigated and largely extended in the palliation practice.
